# Supplementary material for: Splice-Junction-Based Mapping of Alternative Isoforms in the Human Proteome
Source: Cell Rep. Author manuscript; Available in PMC 2020 Jan 15. (PMC6961840; doi:10.1016/j.celrep.2019.11.026)
Supplement: 2 [file NIHMS1546469-supplement-2.zip › DF1/PXD010154/Pancreas/Pancreas_2_PPP1R35_AEEQPGLGPGAAGR.pdf]

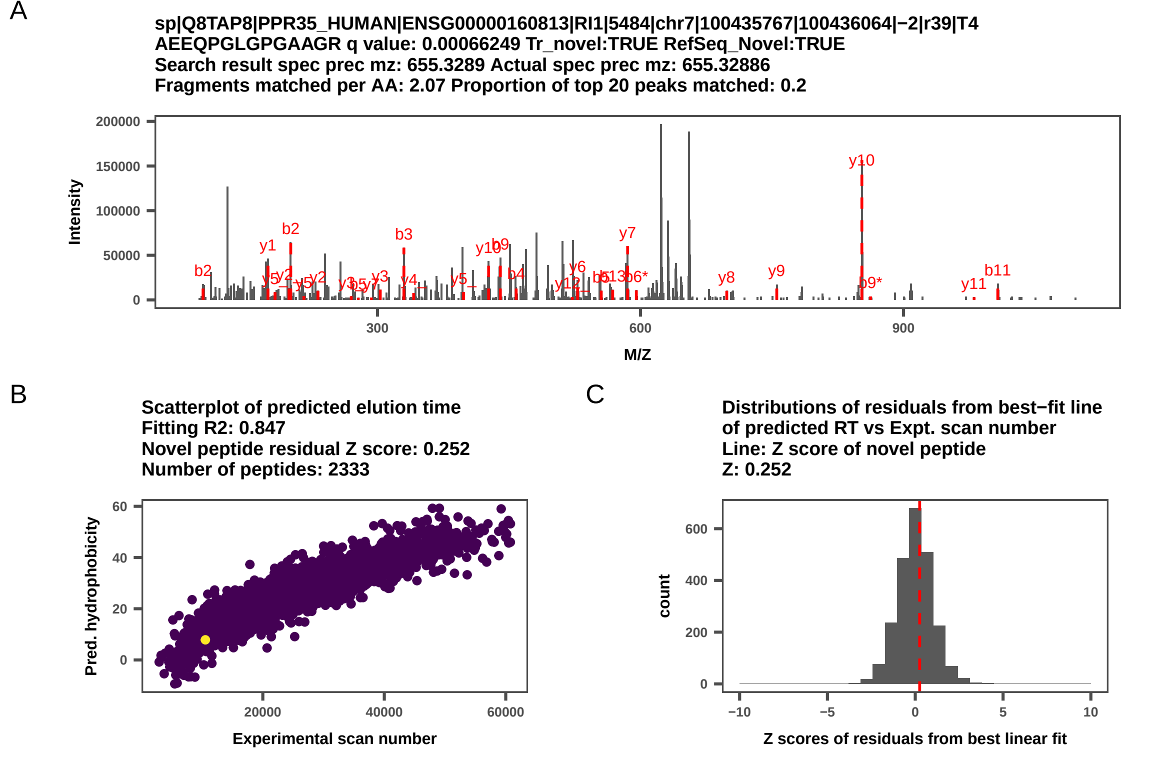
A
sp|Q8TAP8|PPR35_HUMAN|ENSG00000160813|RI1|5484|chr7|100435767|100436064|−2|r39|T4
AEEQPGLGPGAAGR q value: 0.00066249 Tr_novel:TRUE RefSeq_Novel:TRUE
Search result spec prec mz: 655.3289 Actual spec prec mz: 655.32886
Fragments matched per AA: 2.07 Proportion of top 20 peaks matched: 0.2
Intensity
200000
150000
100000
50000
0
M/Z
300
600
900
y10
b2
y1
b2
b3
y7
y6
y8
y9
b9*
y11
b11
b4
y4
y3
B
Scatterplot of predicted elution time
Fitting R2: 0.847
Novel peptide residual Z score: 0.252
Number of peptides: 2333
Pred. hydrophobicity
60
40
20
0
20000
40000
60000
Experimental scan number
C
Distributions of residuals from best−fit line
of predicted RT vs Expt. scan number
Line: Z score of novel peptide
Z: 0.252
count
600
400
200
0
−10
−5
0
5
10
Z scores of residuals from best linear fit
